# QUALITATIVE PERCEPTIONS OF UNDERVACCINATED OLDER ADULTS AND POTENTIAL AVENUES FOR INTERVENTION

**DOI:** 10.1093/geroni/igae098.2475

**Published:** 2024-12-31

**Authors:** Heather Fuller, Andrea Huseth-Zosel, Bryce Van Vleet, Teri Undem

**Affiliations:** North Dakota State University, Fargo, North Dakota, United States; North Dakota State University, Fargo, North Dakota, United States; North Dakota State University, Fargo, North Dakota, United States; North Dakota State University, Fargo, North Dakota, United States

## Abstract

Though older adults typically have relatively high vaccination rates, some older adults are reluctant to receive recommended vaccinations, a trend that is increasing in recent years. Vaccine hesitancy is not well understood among older adults but could help pinpoint areas of intervention to potentially reduce concerns and improve health outcomes. This study qualitatively examines vaccination decision-making processes among older adults (aged 65+) in North Dakota. Qualitative, semi-structured phone interviews focused on access, knowledge, and decisions about vaccines were conducted with 51 older adults between November 2022-January 2023. Of those participants, 24 were selected for the current study due to lacking 33% or more of recommended vaccines for this age group (i.e., influenza, shingles, pneumococcal, COVID-19). Interview transcripts were inductively coded to identify themes related to perceptions of acceptance and hesitancy with making decision about vaccines. Emergent themes included: skepticism, uncertainty of decisions, regret at being misled/fooled, defiance, and confidence in decisions. Within each theme, transcripts were examined to determine potential intervention approaches. To address skepticism, reduction of propaganda campaigns was identified. Creative educational and outreach interventions that emphasize consistent messaging were identified to address both uncertainty and regret. To address defiance and confidence in decisions, interventions should strategically utilize trusted information sources including physicians, pharmacists, and the church while targeting informed, independent decision-making. Findings contribute to better understanding the perceptions and decisions of vaccine hesitant older adults and suggest potential focus areas for interventions to potentially improve vaccination rates and reduce health risks for this population.

